# Prevalence of insomnia and hypnotic use in Norwegian patients visiting their general practitioner

**DOI:** 10.1093/fampra/cmac103

**Published:** 2022-09-19

**Authors:** Sunniva Torsvik, Bjørn Bjorvatn, Knut Eirik Eliassen, Ingeborg Forthun

**Affiliations:** Department of Global Public Health and Primary Care, University of Bergen, Bergen, Norway; Department of Global Public Health and Primary Care, University of Bergen, Bergen, Norway; Norwegian Competence Center for Sleep Disorders, Haukeland University Hospital, Bergen, Norway; Department of Global Public Health and Primary Care, University of Bergen, Bergen, Norway; Department of Global Public Health and Primary Care, University of Bergen, Bergen, Norway

**Keywords:** chronic insomnia, epidemiology, general practice, hypnotics, insomnia disorder, sleep

## Abstract

**Background:**

Sleep problems are common in the general population, but there are few studies on the prevalence of sleep problems and hypnotic use among patients in general practice.

**Objectives:**

To estimate the prevalence of insomnia (based on the Diagnostic and Statistical Manual of Mental Disorders [DSM], version 5), self-reported sleep problems and hypnotic use among patients in general practice, and explore whether the prevalence depended on patient characteristics.

**Methods:**

A cross-sectional study with questionnaire data collected by 114 final-year medical students while deployed in different general practices in Norway during 2020. A total of 1,848 consecutive and unselected patients (response rate 85.2%) visiting their general practitioners (GPs) completed a one-page questionnaire, that included the validated Bergen Insomnia Scale (BIS), questions on for how long they have had a sleep problem, hypnotic use, and background characteristics. Associations were estimated using a modified Poisson regression model.

**Results:**

The prevalence of chronic insomnia according to BIS was 48.3%, while 46.9% reported chronic sleep problems (sleep problems of ≥3 months) and 17.8% reported hypnotic use. Females, patients with low compared with higher education, and patients who slept shorter or longer than 7–8 h, had higher risk of chronic insomnia disorder (CID), chronic self-reported sleep problems (CSP), and hypnotic use. The oldest age group (≥65 years) had lower risk of chronic insomnia compared with the youngest (18–34) but twice the probability of hypnotic use.

**Conclusions:**

CID, CSP, and hypnotic use were prevalent among patients visiting their GP. Insomnia can be effectively treated and deserves more attention among GPs.

**Key messages**

- There are few prevalence studies on sleep problems in general practice.
- The diagnostic criteria for insomnia have been changed.
- Prevalence of chronic insomnia disorder was 48% among patients visiting their GPs.
- The prevalence of hypnotic use was 18%.
- Insomnia can be effectively treated and deserves more attention among GPs.

## Introduction

Sleep problems are common in the general population, and insomnia is the most prevalent sleep disorder with a reported prevalence of about 10%.^1^ The amount of people suffering from insomnia symptoms is even higher. Insomnia is often comorbid with chronic psychiatric and somatic disorders,^2–5^ and found to be a significant risk factor for cardiovascular disease,^6,7^ anxiety,^8^ and depression.^8,9^ Although insomnia may be caused or maintained by other comorbid disorders, it is considered an independent diagnosis that requires targeted treatment. Cognitive behavioural therapy for insomnia (CBT-I) is the recommended first-line treatment for chronic insomnia disorder (CID), although hypnotic medication is still the most used therapeutic modality with an estimated prevalence of 11% in the Norwegian general population.^1,10^

Since insomnia tend to coexist with other psychiatric or somatic diseases,^2–5^ it is likely that the prevalence of insomnia and hypnotic use is higher among patients visiting their general practitioner (GP) compared with the general population. There are, however, few studies exploring this.^11–19^ The present study builds on a similar Norwegian study from 2014 that reported a prevalence of chronic insomnia and hypnotic use of 50% and 16%, respectively, in patients in general practice.^11^ In that study, the diagnostic criteria for chronic insomnia were based on the Diagnostic and Statistical Manual of Mental Disorders, version 4 (DSM-IV).^20^ In the new version of the DSM (DSM-5), the qualifying duration of chronic insomnia was increased from ≥1 to ≥3 months and non-restorative sleep was removed as criteria.^21^ As a result, this version better distinguishes between insomnia and other sleep disorders than DSM-IV.^20^ Similar revisions have been made in the International Classification of Diseases, version 11 (ICD-11).^22^ There is a scarcity of studies on insomnia in general practice using the revised diagnostic criteria. A recent study among primary care patients from Switzerland, reported a prevalence of chronic insomnia based on the DSM-5 criteria of 11%.^12^ This is considerably lower than the results from the study by Bjorvatn et al.,^11^ and indicates that more studies on the prevalence of insomnia using the new criteria are needed. Updated data on the prevalence of insomnia and hypnotic use are needed in terms of raising the GPs’ awareness of the importance of sleep problems in their patient population.

In the general population, the prevalence of insomnia and hypnotic use is usually found to be higher among females than males, and to increase with age.^2,23^ Interestingly, some studies in general practice have found insomnia to be less common in older age groups compared with younger age groups.^11,13^ Insomnia and hypnotic use have been reported to be more common in people with lower education and in people without children living at home,^23–25^ and have also been linked to sleep-related parameters. Evening types experience more insomnia symptoms^26,27^ and have a higher hypnotic use compared with other circadian types.^27^ A higher prevalence of insomnia symptoms has also been found in both individuals with shorter and longer sleep duration than 8 h.^28^

With this as a backdrop, our main objective was to estimate the prevalence of CID based on the DSM-5 diagnostic criteria among patients in general practice, in addition to the prevalence of self-reported sleep problems and hypnotic use, and whether the prevalence depended on patient characteristics such as sex, age, education, having children living at home, sleep duration, and circadian preference (morningness–eveningness).

## Methods

In Norway, GPs have since 2001 been organized in a patient list system in which each citizen has the legislated right to be included on a GP’s list.

### Selection of study subjects

During the last year of medical school at the University of Bergen, all students are deployed in different general practices in Western Norway for 6 weeks. While deployed, the students were asked, on a voluntary basis, to approach 20 unselected consecutive patients 18 years or older, asking if they would complete a one-page questionnaire while waiting for the consultation with the doctor. A total of 153 medical students were asked to take part, potentially resulting in 3,060 responses. No power calculation was conducted. We anticipated that about 2,300 patients would answer the questionnaire with an expected response rate of 75%, based on experience from similar data collections in previous years.^29^ The patients were asked regardless of past and current medical history and the contact reason was not registered. The first part of the questionnaire included general information about the purpose of the study, and specified that participation was anonymous and voluntary. Data collection was performed during the spring and fall semesters of 2020.

### Measurements

The questionnaire included questions about sleep, infections, and medication use (antibiotics and/or hypnotics). Only data from the sleep-related questions are presented here. The patients were also asked about their age, sex, highest attained education, and if they had children living at home.

Insomnia was measured with the validated Bergen Insomnia Scale (BIS).^30^ BIS consists of 6 items and was developed based on the diagnostic criteria for insomnia according to the DSM-IV.^20^ In the present study, the scale was adapted according to the updated DSM-5 diagnostic criteria.^21^ The items are scored along an 8-point scale indicating the number of days per week during the past 3 months for which a specific insomnia symptom is experienced (0–7 days). The first 3 items reflect sleep onset, maintenance, and early morning awakening insomnia, and the last 2 focus on daytime impairment and being dissatisfied with current sleep (see Appendix). CID was defined as scoring 3 days per week or more on at least one of the first 3 items as well as 3 or more on at least one of the 2 latter items. Cronbach’s *α* for BIS was 0.88 in the present sample.

BIS measures symptoms of insomnia but the patients are not specifically asked if they experience having sleep problems. For this reason, we included an additional question on sleep problems (“For how long have you experienced sleep problems?”), measured with a 4-point scale (“do not have sleep problems,” “less than 3 months,” “3 months to a year,” or “more than a year”). Based on this question we created a variable for chronic self-reported sleep problems (CSP), defined as sleep problems that had lasted 3 months or more, making the variable comparable with CID according to BIS. Hypnotic use (“Do you use hypnotics?”) was self-reported on a 5-point scale (“no,” “sometimes,” “1–2 days per week,” “3–6 days per week,” or “daily”) but dichotomized as “no” and “yes” (included “sometimes” or more frequent). Morningness–eveningness (“Are you a morning [lark] or evening [owl] type?”) was self-reported on a 5-point scale (“definitively a morning type,” “more a morning than an evening type,” “neither a morning nor an evening type,” “more an evening than a morning type,” or “definitely an evening type”). Based on this question, we created a new variable with the first 2 categories included as “morning type (lark),” the middle category as “neither a morning nor an evening type,” and the last 2 categories as “night type (owl).” The respondents were asked to report sleep duration using 5 categories (“less than 6 h,” “6–7 h,” “7–8 h,” “8–9 h,” “more than 9 h”).

### Statistical analyses

We compared CID, CSP, and hypnotic use by sex (“male,” “female”), age (“18–34,” “35–49,” “50–64,” “≥65” years), education (“primary and lower secondary education,” “upper secondary education,” “vocational school,” “higher education”), children living at home (“yes,” “no”), morningness–eveningness, and sleep duration using Pearson’s chi-square tests. Furthermore, we used a modified Poisson model to estimate crude and adjusted relative risks (RRs) with 95% confidence intervals (CIs) for the association between each predictor and each of the 3 outcome variables (CID, CSP, and hypnotic use).^31^ This model has shown to yield very similar results to the conventional binomial estimation procedure and is the recommended approach in cases where one wants to estimate RR, but the log binomial model fails to converge.^31,32^ In the adjusted analyses, we adjusted for sex, age, education, children living at home, and morningness–eveningness. We conducted sensitivity analyses in which we checked for possible differences in the results between the spring and fall semester. We found no association between semester of data collection and the outcome variables, and this variable did not affect the association between the other variables and the outcomes. Stata (version SE 16.1) was used for all statistical analyses.

## Results

**Fig. 1.**
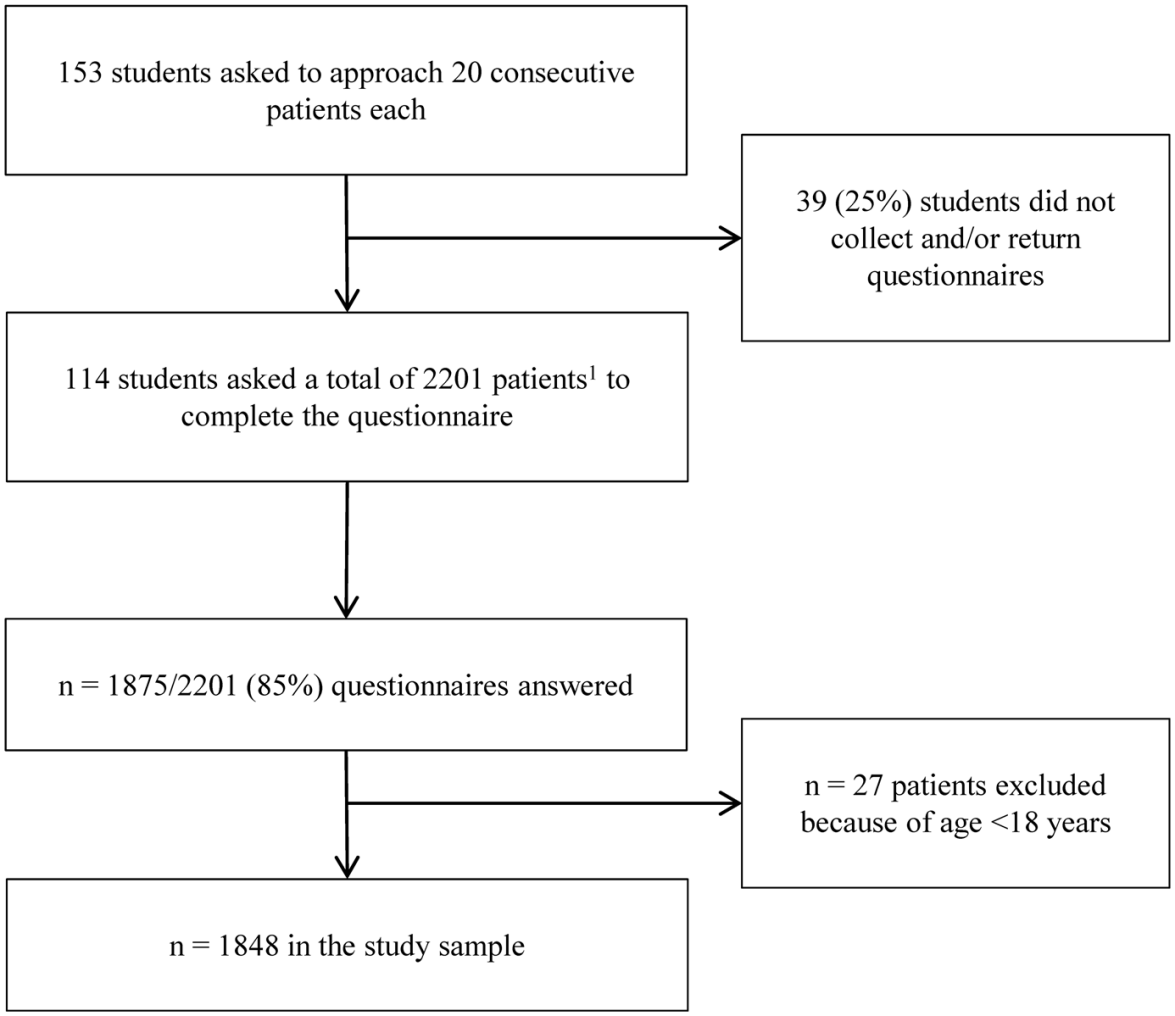
Flow chart of data collection. ^1^Some of the students asked fewer than 20 patients to participate in the study, hence the total number of patients asked are lower than the expected number of 114 students × 20 questionnaires = 2,280 patients asked.

Of a total of 153 students, 114 collected data for this study. The total number of collected questionnaires was 2,201, of which 1,875 were answered, resulting in a response rate of 85.2%. Excluding patients under 18 years of age resulted in a final sample of 1,848 patients (Fig. 1). Females constituted 60.6% of the study sample (Table 1). Mean age was 51.8 years, 38.0% had a higher education, and 33.3% had children living at home. In terms of sleep duration, 21.0% reported sleeping less than 6 h, while only 2.0% slept more than 9 h.

**Table 1.** Patient characteristics and prevalence of CID among 1,848 patients in Western Norway visiting their GP during 2020.

	*n*	%
Sex, *n* = 1,812 (82.3%)^a^		
Female	1,098	60.6
Male	714	39.4
Age in years, *n* = 1,779 (80.8%)^a^		
18–34	381	21.4
35–49	409	23.0
50–64	483	27.2
≥65	506	28.4
Education, *n* = 1,705 (77.5%)^a^		
Primary and lower secondary education	189	11.1
Upper secondary education	505	29.6
Vocational school	363	21.3
Higher education	648	38.0
Children living at home, *n* = 1,650 (75.0%)^a^		
Yes	550	33.3
No	1,100	66.7
CID, DSM-5 criteria, *n* = 1,716 (78.0%)^a^		
Yes	829	48.3
No	887	51.7
For how long have you experienced sleep problems?, *n* = 1,769 (80.4%)^a^		
Do not have sleep problems	826	46.7
Less than 3 months	114	6.4
3 months to 1 year	140	7.9
More than a year	689	39.0
Do you use hypnotics?, *n* = 1,804 (82.0%)^a^		
No	1,482	82.2
Sometimes	141	7.8
1–2 days per week	24	1.3
3–6 days per week	47	2.6
Daily	110	6.1
Sleep duration, *n* = 1,796 (81.6%)^a^		
Less than 6 h	377	21.0
6–7 h	828	46.1
7–8 h	448	24.9
8–9 h	108	6.0
More than 9 h	35	2.0
Are you a morning type (lark) or an evening type (owl)?, *n* = 1,791 (81.4%)^a^		
Definitely a morning type	327	18.3
More a morning than an evening type	420	23.5
Neither a morning nor an evening type	372	20.8
More an evening than a morning type	449	25.1
Definitely an evening type	223	12.5

^a^Response rate of the specific question.

CID based on the DSM-5 criteria was present in 48.3% of the patients (95% CI 45.9%–50.7%) (Table 1). In comparison, 46.9% reported having CSP (95% CI 44.5%–49.2%). Of all the patients with CSP, 80.1% met the diagnostic criteria for CID, whereas 78.3% of the patients having CID had CSP. Hypnotic use was reported by 17.8% (95% CI 16.1%–19.7%). Furthermore, 26.0% of the patients with CID and 32.3% of the patients with CSP used hypnotics. In the study sample, only 9.6% reported no sleep problems at all, defined as not having CSP, not using hypnotics, and not reporting any days during the last 3 months with more than 30 min sleep onset, wake after sleep onset, or early morning awakening.

There was a higher prevalence of CID, CSP, and hypnotic use among females compared with males (Table 2). CID, CSP, and hypnotic use also varied significantly by age, education, and sleep duration. Among patients sleeping less than 6 h per day, as many as 81.6% met the criteria for CID.

**Table 2.** Prevalence of CID, CSP, and hypnotic use among 1,848 patients visiting their GPs in Western Norway during 2020, by independent variables (sex, age, education, children living at home, sleep duration, and morningness–eveningness)^a^.

	CID^b^	CSP^c^	Hypnotic use
Sex (%)	*P* < 0.001	*P* = 0.003	*P* = 0.001
Female	53.2	49.8	20.0
Male	40.6	42.5	14.1
Age, years (%)	*P* < 0.001	*P* = 0.001	*P* < 0.001
18–34	56.3	40.7	11.1
35–49	45.1	45.0	14.2
50–64	52.0	53.9	16.4
≥65	40.1	46.1	25.7
Education (%)	*P* = 0.011	*P* = 0.004	*P* < 0.001
Primary and lower secondary education	53.6	52.2	29.7
Upper secondary education	52.1	49.4	17.2
Vocational school	49.4	50.4	18.9
Higher education	43.2	41.2	13.7
Children living at home (%)	*P* = 0.583	*P* = 0.017	*P* < 0.001
Yes	49.2	42.1	11.1
No	47.7	48.5	20.8
Sleep duration (%)	*P* < 0.001	*P* < 0.001	*P* < 0.001
Less than 6 h	81.6	78.7	26.8
6–7 h	44.6	43.1	14.7
7–8 h	28.0	26.7	13.4
8–9 h	37.8	37.7	20.4
More than 9 h	55.9	74.3	25.7
Morning type (lark) or evening type (owl) (%)	*P* = 0.007	*P* = 0.012	*P* = 0.193
Morning type	43.8	42.8	16.0
Neither morning nor evening type	50.0	51.4	19.1
Evening type	52.1	49.0	19.4

^a^ *P* value from chi-square test.

^b^Based on the BIS after the DSM-5 criteria for CID.

^c^Defined as self-reported sleep problems lasting 3 months or more.

In the regression analyses, females had 28% higher risk of CID (adjusted relative risk [aRR] 1.28, 95% CI 1.14–1.44) and 50% higher risk of hypnotic use compared with males (aRR 1.50, 95% CI 1.18–1.90) (Table 3). Patients aged 35–49 (aRR 0.80, 95% CI 0.68–0.93) and ≥65 (aRR 0.73, 95% CI 0.62–0.86) had lower risk of CID compared with the reference group (18–34 years). However, patients aged ≥65 years had more than a doubled risk of hypnotic use compared with the youngest age group (aRR 2.08, 95% CI 1.47–2.95). The risk of CID, CSP, and hypnotic use was higher in patients in all educational groups compared with those with higher education. For hypnotic use, the highest increase in risk was found for patients with primary and lower secondary education (aRR 1.75, 95% CI 1.26–2.42). Patients with children living at home had lower risk of hypnotic use compared with patients without children at home (aRR 0.64, 95% CI 0.44–0.92).

**Table 3.** Crude and adjusted^a^ RR with 95% CI of CID, CSP, and hypnotic use by independent variables, among patients visiting their GPs in Western Norway during 2020.

Independent variables	CID		CSP		Hypnotic use	
	Crude	Adjusted^a^	Crude	Adjusted^a^	Crude	Adjusted^a^
Sex						
Female	1.31 (1.18–1.46)	1.28 (1.14–1.44)	1.17 (1.05–1.30)	1.19 (1.06–1.33)	1.42 (1.14–1.77)	1.50 (1.18–1.90)
Male	1 (Ref.)	1 (Ref.)	1 (Ref.)	1 (Ref.)	1 (Ref.)	1 (Ref.)
Age, years						
18–34	1 (Ref.)	1 (Ref.)	1 (Ref.)	1 (Ref.)	1 (Ref.)	1 (Ref.)
35–49	0.80 (0.70–0.92)	0.80 (0.68–0.93)	1.11 (0.94–1.30)	1.18 (1.00–1.41)	1.27 (0.88–1.85)	1.48 (0.98–2.24)
50–64	0.92 (0.81–1.04)	0.95 (0.83–1.09)	1.32 (1.14–1.54)	1.32 (1.13–1.55)	1.47 (1.04–2.09)	1.46 (1.01–2.09)
≥65	0.71 (0.61–0.82)	0.73 (0.62–0.86)	1.13 (0.97–1.33)	1.12 (0.94–1.33)	2.30 (1.67–3.18)	2.08 (1.47–2.95)
Education						
Primary and lower secondary education	1.24 (1.04–1.47)	1.31 (1.10–1.56)	1.27 (1.07–1.50)	1.24 (1.04–1.48)	2.17 (1.61–2.91)	1.75 (1.26–2.42)
Upper secondary education	1.21 (1.06–1.37)	1.19 (1.05–1.35)	1.20 (1.05–1.36)	1.19 (1.04–1.36)	1.25 (0.95–1.65)	1.27 (0.96–1.69)
Vocational school	1.14 (0.99–1.31)	1.21 (1.05–1.41)	1.22 (1.06–1.41)	1.24 (1.07–1.44)	1.37 (1.03–1.84)	1.30 (0.96–1.75)
Higher education	1 (Ref.)	1 (Ref.)	1 (Ref.)	1 (Ref.)	1 (Ref.)	1 (Ref.)
Children living at home						
Yes	1.03 (0.93–1.15)	1.01 (0.88–1.14)	0.87 (0.77–0.98)	0.87 (0.75–1.00)	0.53 (0.41–0.70)	0.64 (0.44–0.92)
No	1 (Ref.)	1 (Ref.)	1 (Ref.)	1 (Ref.)	1 (Ref.)	1 (Ref.)
Sleep duration						
Less than 6 h	2.91 (2.48–3.42)	3.00 (2.52–3.58)	2.95 (2.50–3.47)	3.01 (2.51–3.62)	2.01 (1.50–2.69)	2.29 (1.66–3.16)
6–7 h	1.59 (1.34–1.89)	1.61 (1.34–1.94)	1.61 (1.36–1.92)	1.70 (1.40–2.06)	1.10 (0.83–1.47)	1.22 (0.88–1.68)
7–8 h	1 (Ref.)	1 (Ref.)	1 (Ref.)	1 (Ref.)	1 (Ref.)	1 (Ref.)
8–9 h	1.35 (1.00–1.81)	1.40 (1.03–1.91)	1.41 (1.06–1.89)	1.47 (1.08–2.01)	1.53 (0.98–2.37)	1.62 (1.00–2.62)
More than 9 h	1.99 (1.42–2.79)	1.77 (1.24–2.53)	2.78 (2.17–3.57)	2.74 (2.08–3.60)	1.93 (1.05–3.55)	1.99 (1.00–3.97)
Morning type (lark) or evening type (owl)						
Morning type	0.88 (0.76–1.00)	0.90 (0.78–1.05)	0.83 (0.73–0.95)	0.77 (0.67–0.89)	0.84 (0.64–1.09)	0.78 (0.58–1.04)
Neither morning nor evening type	1 (Ref.)	1 (Ref.)	1 (Ref.)	1 (Ref.)	1 (Ref.)	1 (Ref.)
Evening type	1.04 (0.92–1.18)	1.04 (0.91–1.20)	0.95 (0.84–1.08)	0.95 (0.83–1.09)	1.02 (0.78–1.32)	1.11 (0.84–1.48)

^a^Adjusted for sex, age, education, children living at home, and morningness–eveningness. Adjusted analyses included those with complete information on all variables in the model.

For the sleep-related variables, we found that morning types had lower risk of CSP (aRR 0.77, 95% CI 0.67–0.89) compared with the reference group (neither morning nor evening type) (Table 3). A strong association was found between sleep duration and all 3 outcome variables. Most evident was a higher risk of CID and CSP for patients sleeping less than 6 h (aRR for CID 3.00, 95% CI 2.52–3.58, aRR for CSP 3.01, 95% CI 2.51–3.62) and for those who slept more than 9 h per day (aRR for CID 1.77, 95% CI 1.24–2.53, aRR for CSP 2.74, 95% CI 2.08–3.60) compared with the reference group of 7–8 h. Those who slept less than 6 h (aRR 2.29, 95% CI 1.66–3.16) or more than 9 h per day (aRR 1.99, 95% CI 1.00–3.97) also had higher risk of hypnotic use.

## Discussion

We found a high prevalence of both CID and CSP (sleep problems of ≥3 months) among patients visiting their GP. The prevalence of CID was 48.3%, based on the BIS using the DSM-5 diagnostic criteria. A similar number of patients, 46.9%, had CSP. Hypnotic use was reported by 17.8%.

In previous studies, the reported prevalence of chronic insomnia in patients in general practice has varied, partly depending on the different methodological approaches and diagnostic criteria used.^11–19^ The findings of the present study are consistent with the results from the study by Bjorvatn et al.,^11^ who used the same insomnia scale (BIS) and method of selecting patients, but used the less strict DSM-IV criteria for chronic insomnia. In that study, the prevalence of chronic insomnia, self-reported sleep problems (“Do you experience sleep problems?”), and hypnotic use was 53.6%, 55.8%, and 16.2%, respectively.^11^ Another study among primary care patients in Malaysia, with a similar study setting as the present study and using the DSM-IV criteria, found an insomnia prevalence of 28.6%.^18^ In a more recent study from Switzerland,^12^ primary care physicians (PCPs) were asked to collect data about insomnia symptoms and treatment on a consecutive group of their adult patients using a standardized paper form. The authors found an insomnia prevalence of 11% based on the DSM-5 criteria,^12^ which is notably lower than the prevalence in the present study. Discrepancy in prevalence of insomnia between these two studies may in part be explained by differences in survey methodology and assessment of insomnia symptoms. Unlike in our study, the study from Switzerland did not include an unselected sample of patients and data were collected by the PCPs and not based on self-report. In the Swiss study, the PCPs could choose not to discuss sleep with a patient if they felt that was inappropriate, and symptoms of insomnia were only registered for patients who reported to have current sleep complaints. In the present study, all patients completed the BIS, regardless of their own subjective experience of sleep problems. More studies are needed to establish the effects of the changed diagnostic criteria on the prevalence of CID.

There was a clear concurrence between CID and CSP. However, around 20% of those with CSP did not fulfil the diagnostic criteria for CID, and vice versa. The patients who reported to have a sleep problem, but did not meet the diagnostic criteria for insomnia, might suffer from another sleep disorder, e.g. circadian rhythm sleep–wake disorders or restless legs.^5^ Furthermore, the patients with CID according to BIS, but not with CSP, may not consider or recognize their insomnia symptoms as a significant problem. In the present study, 17.8% of the total sample, and about 30% of all patients with CID or CSP, reported hypnotic use. In comparison, in the last decade, about 8% of the general adult population has filled at least one prescription of hypnotics each year according to the Norwegian Prescription Database.^33^ The registry does not report on actual use, but in two survey-based Norwegian studies, 11.1% and 7.9%, respectively, reported to use hypnotics at least once in the last month.^10,23^ Short-term beneficial effects of hypnotic use on CID have been found, but long-term use is in general not recommended due to drug tolerance, risk of addiction, and other adverse effects.^2^ CBT-I—the recommended treatment—has been found to be highly effective, also when delivered in primary care.^34^ Non-pharmacological approaches are preferred by patients,^23^ but CBT-I is underprescribed by GPs,^1,12^ including in Norway. This could be due to both resource constraints, lack of training, and knowledge.

In accordance with other studies, we found that CID and hypnotic use were more common among females than males.^2,14,23,24^ Insomnia was prevalent in all age groups, each with a prevalence of at least 40%. Interestingly, the youngest age group (18–34 years) presented the highest risk of CID, whereas other studies in general practice have found the insomnia prevalence to increase with age.^12,14^ Our results are however in accordance with Bjorvatn et al.,^11^ who found a higher prevalence of insomnia in younger patients, and a study conducted among 955 primary care patients in New Zealand.^13^ Younger patients may more often seek their GP when they are ill, while older patients to a greater extent may undergo regular checkups or visit their GPs for other reasons without being subjectively ill. This could cause a selection effect and affect the prevalence of comorbid insomnia. The study from New Zealand found a higher prevalence of insomnia due to psychological problems among younger patients, but the youngest age group also had a higher prevalence of primary insomnia.^13^ Increasing rates of insomnia symptoms over time have also been found among Norwegian college and university students (18–35 years old).^35^ In the present study we found that although patients 65 years or older had the lowest risk of CID, they had the highest prevalence of hypnotic use (25.7%). Higher rates of hypnotic use among the oldest age group may possibly explain the lower prevalence of CID in this group. This is, however, difficult to assess due to the cross-sectional design of the present study. The lower prevalence of hypnotic use in the youngest age group may be due to few cases of multimorbidity in this age group as patients with multiple chronic diseases are prescribed more hypnotic and/or anxiolytic medication,^36^ or be due to the GPs having a higher threshold for prescribing hypnotics to younger adults.^37^ The probability of hypnotic use was also lower among patients with children compared with those without children. Having children might be an indicator of better health, or patients with children at home might be more reluctant to use hypnotics for their sleep problems due to possible side effects and family obligations.

Patients with both shorter and longer sleep duration than 7–8 h had a higher risk of CID and CSP. Furthermore, patients with sleep duration less than 6 h had an increased risk of hypnotic use. Insomnia with objective short sleep duration (sleeping less than 6 h) has been suggested as the most biologically severe phenotype of the disorder, and is associated with an increased risk of hypertension, type 2 diabetes, and mortality.^38,39^ A Norwegian follow-up cohort study among 6,599 working adults (40–45 years old) in the general population found that people with insomnia had shorter average sleep duration compared with people without insomnia.^40^ An association between long sleep duration and insomnia symptoms has been less reported. However, in an American study with a nationally representative sample of 1,004 adults, a higher prevalence of insomnia symptoms was found in both individuals with shorter and longer sleep duration than 8 h.^28^

### Strengths and limitations

The present study has important strengths and limitations. The study sample was relatively large and included an unselected group of patients from general practice. Patients experiencing sleep problems might have been more eager to participate in the study, which in turn could introduce selection bias. However, the high response rate of 85.2% indicates that the results are likely generalizable to patients in general practice. Other ways to collect data from general practice, for instance by involving the GPs directly or by using trained personnel, may have resulted in different response rates. Another strength was the high internal consistency of the BIS, with a Cronbach’s *α* of 0.88. Even so, the specificity of BIS may be rather low and could measure other sleep disorders than insomnia. Furthermore, BIS is validated based on the DSM-IV criteria for chronic insomnia,^30^ and has not been validated based on the DSM-5 criteria used in the present study. The data were based on self-report, and no clinical assessment (or supplementary tests) required for a definite insomnia diagnosis were conducted. We do not know the reason for the patients’ consultation with their GP, and whether they sought advice or treatment for a sleep problem. No exclusion criteria for other sleep disorders (e.g. obstructive sleep apnoea syndrome, restless legs, circadian rhythm sleep–wake disorders) were implemented. Objective measurements of sleep duration by polysomnography or other methods were not considered feasible to implement in this study setting. Moreover, the patients were not asked how long they had used hypnotics, what type they used and for what reason they were prescribed.

Out of 153 medical students, only 114 (74.5%) returned questionnaires. This resulted in a lower sample than anticipated (final sample of 1,848 compared with an expected sample of about 2,300 patients). The questionnaires were collected during the spring and fall of 2020. We believe that the limited deliverance rate among students was partly due to the COVID-19 pandemic, which probably affected the students’ opportunity to see patients. The University campus was also closed for several months, and as a result the students were unable to return the questionnaires immediately following their deployment period. Some students may have lost or forgotten to return the questionnaires. We do not have information about the students who did not return questionnaires, but do not believe there were any systematic differences between them and those who did. Furthermore, we do not think that the limited deliverance rate among students affected the response rate among patients or had any substantial effect on the observed results. The COVID-19 pandemic did, however, likely affect the selection of patients visiting their GP, and consequently may have influenced the prevalence and associations found in the present study. In turn, this could influence the generalizability of the present findings.^41^ Most of the data collection in the spring semester occurred during the initial lockdown in Norway, while fewer restrictions were in place in the fall semester. The restrictions, especially during lockdown, may have increased many patients’ threshold to visit their GP. Furthermore, almost all GPs in Norway implemented video consultations in the early phase of the pandemic while the questionnaires were only handed out to those who met to their appointment in person. When comparing background characteristics in the present study with Bjorvatn et al.^11^ (age and sex), we found a higher percentage of patients in the oldest age group in the present study. This difference in age distribution may be due to video consultations being more common among younger patients. At the same time, the prevalence estimates reported in the present study correspond well with the previous study by Bjorvatn et al.

## Conclusions

CID was present in 48.3% of the patients visiting their GP, 46.9% had CSP, and 17.8% reported use of hypnotics. The results from the present study confirm that both insomnia and hypnotic use are highly prevalent among patients in general practice. Awareness among GPs on diagnostic evaluation and treatment of insomnia is important.

## Supplementary Material

cmac103_suppl_Supplementary_Appendix

cmac103_suppl_Supplementary_Questionnaire

cmac103_suppl_Supplementary_Checklist

## Data Availability

The data underlying this article will be shared on reasonable request to the corresponding author.
