# Supplementary material for: Prevalence of insomnia and hypnotic use in Norwegian patients visiting their general practitioner
Source: Fam Pract. 2022 Sep 19;40(2):352–9. doi: 10.1093/fampra/cmac103 (PMC10047630; doi:10.1093/fampra/cmac103)
Supplement: cmac103_suppl_Supplementary_Appendix [file cmac103_suppl_supplementary_appendix.docx]

**Appendix**

*Bergen Insomnia Scale^1^*

The questionnaire below contains six questions relating to sleep and tiredness. Please circle the alternative (number of days per week) that suits you best. 0 means no days during the course of a week, 7 means every day during the course of a week.

|  |  | Number of days per week |
| --- | --- | --- |
| 1. | During the past three months, how many days a week has it taken you more than 30 minutes to fall asleep after the light was switched off? | 0 1 2 3 4 5 6 7 |
| 2. | During the past three months, how many days a week have you been awake for more than 30 minutes between periods of sleep? | 0 1 2 3 4 5 6 7 |
| 3. | During the past three months, how many days a week have you woken up more than 30 minutes earlier than you wished without managing to fall asleep again? | 0 1 2 3 4 5 6 7 |
| 4. | During the past three months, how many days a week have you felt that you have not had enough rest after waking up? | 0 1 2 3 4 5 6 7 |
| 5. | During the past three months, how many days a week have you been so sleepy/tired that it has affected you at school/work or in your private life? | 0 1 2 3 4 5 6 7 |
| 6. | During the past three months, how many days a week have you been dissatisfied with your sleep? | 0 1 2 3 4 5 6 7 |

^1^Bergen Insomnia Scale (BIS) consists of six items and was developed based on the diagnostic criteria for insomnia according to the Diagnostic and Statistical Manual of Mental Disorders, 4th edition (DSM-IV). In the present study the scale was adapted according to the updated DSM-5 diagnostic criteria, where non-restorative sleep was removed as a criteria (item four). Chronic insomnia disorder according to the DSM-5 criteria was defined as scoring 3 days per week or more on at least one of the first three items as well as 3 or more on at least one of the two latter items.
