# Supplementary material for: Prevalence of insomnia and hypnotic use in Norwegian patients visiting their general practitioner
Source: Fam Pract. 2022 Sep 19;40(2):352–9. doi: 10.1093/fampra/cmac103 (PMC10047630; doi:10.1093/fampra/cmac103)
Supplement: cmac103_suppl_Supplementary_Questionnaire [file cmac103_suppl_supplementary_questionnaire.docx]

Age: _____years

Sex:  female  male

Birth country:  Norway  Other country in Europe  Asia  Africa  America  Oceania

Highest attained education: primary/lower secondary  upper secondary vocational  university

Do you have children living at home?  no  yes, 0-2 years  yes, 3-6 years  yes, 7-12 years  yes, 13-18 years

| PART 1: Have you had any infections during the **last 3 months** (Check all that apply): | | | | |
| --- | --- | --- | --- | --- |
| \|  \| 0 times \| 1 time \| 2 times \| 3 times \| More than 3 times \| \| --- \| --- \| --- \| --- \| --- \| --- \| \| Common cold \|  \|  \|  \|  \|  \| \| Infection of throat, otitis or sinusitis \|  \|  \|  \|  \|  \| \| Pneumonia/bronchitis \|  \|  \|  \|  \|  \| \| Eye infection \|  \|  \|  \|  \|  \| \| Gastrointestinal infection with vomit or diarrhea \|  \|  \|  \|  \|  \| \| Urinary tract infection \|  \|  \|  \|  \|  \| \| Skin infection \|  \|  \|  \|  \|  \| \| Other infection \|  \|  \|  \|  \|  \|   Do you experience infections more often than others of your age?  no  a little more  somewhat more  much more  very much more  Have you used antibiotics in the last 3 months?  no  yes, one prescription  yes, two prescriptions  yes, three or more prescriptions |  |  |  |  |

PART 2: The questionnaire below contains six questions relating to sleep and tiredness. Please circle the alternative (number of days per week) that suits you best. 0 means no days during the course of a week, 7 means every day during the course of a week.

| During the past 3 months. how many days a week has it taken you more than 30 minutes to fall asleep after the light was switched off? | 0 1 2 3 4 5 6 7 |
| --- | --- |
| During the past 3 months, how many davs a week have you been awake for more than 30 minutes between periods of sleep? | 0 1 2 3 4 5 6 7 |
| During the past 3 months, how many days a week have you awakened more than 30 minutes earlier than you wished without managing to fall asleep again? | 0 1 2 3 4 5 6 7 |
| During the past 3 months, how many days a week have vou felt that vou have not had enough rest after waking up? | 0 1 2 3 4 5 6 7 |
| During the past 3 months, how many days a week have you been so sleepy/tired that it has affected you at school/work or in your private life? | 0 1 2 3 4 5 6 7 |
| During the past 3 months, how many days a week have you been dissatisfied with your sleep? | 0 1 2 3 4 5 6 7 |

For how long have you had a sleep problem?  do not have a sleep problem  less than 3 months  3 months-1 year  more than 1 year

Do you use sleep medication on prescription?  no  sometimes  1-2 days/week  3-6 days/week  daily

Approximately how long do you sleep per day?  less than 6h  6-7h  7-8h  8-9h  more than 9h

Are you a morning type or evening type? Check only one box:

definitely a morning type  more morning than evening type  neither  more evening than morning type

definitely an evening type

**THANK YOU FOR PARTICIPATING!**
